# From gut to heart: *Salmonella* gastroenteritis complicated by myocarditis: a case report

**DOI:** 10.1016/j.clinme.2025.100503

**Published:** 2025-08-20

**Authors:** Orestis Paschalis, Paul Njoku, Amit K.J. Mandal, Constantinos G. Missouris

**Affiliations:** a School of Clinical Medicine, University of Cambridge, Cambridge, United Kingdom; b Departments of Medicine and Cardiology, Wexham Park Hospital, Frimley Health NHS Foundation Trust, Berkshire, United Kingdom; c Department of Clinical Cardiology, University of Nicosia Medical School, Nicosia, Cyprus

**Keywords:** Salmonella, Myocarditis, Gastroenteritis, Cardiac magnetic resonance imaging

## Abstract

Acute myocarditis is an inflammatory condition of the heart muscle, most commonly caused by viral infections. Bacterial myocarditis, particularly due to non-typhoidal *Salmonella* (NTS), is exceptionally uncommon in immunocompetent individuals. We report a rare case of acute myocarditis secondary to *Salmonella* gastroenteritis in a woman in her early 20s, characterised by elevated cardiac biomarkers, positive stool cultures for *Salmonella enterica*, electrocardiographic changes and confirmatory cardiac magnetic resonance imaging. Management was conservative with anti-inflammatory monotherapy without antibiotic treatment, resulting in full clinical recovery with resolution of symptoms and normalisation of cardiac biomarkers. This case highlights a rare presentation of NTS-associated myocarditis and suggests that conservative management may be sufficient in selected cases. It underscores the importance of considering bacterial pathogens, including NTS, in the differential diagnosis of acute myocarditis, even in immunocompetent patients, and highlights the utility of multimodal imaging and individualised management strategies in achieving favourable outcomes in bacterial myocarditis.

## Introduction

Myocarditis presents a spectrum of clinical manifestations, from mild, self-limiting cases to fulminant heart failure and sudden cardiac death. Viral infections, such as those caused by coxsackievirus, adenovirus and parvovirus B19, are the most common aetiologies globally.^1^ Bacterial myocarditis is rare, particularly in immunocompetent individuals, and may be caused by pathogens such as *Staphylococcus aureus, Borrelia burgdorferi* (Lyme disease) or, rarely, non-typhoidal *Salmonella* (NTS).^2^

The pathogenesis involves direct myocardial invasion, immune-mediated inflammation or a combination of both. In bacterial myocarditis, systemic endotoxaemia or bacteraemia often triggers myocardial inflammation rather than direct cardiac infection.^3^ Early diagnosis is key to avoiding complications such as dilated cardiomyopathy and ventricular arrhythmias.

Diagnosis is based on clinical findings, cardiac biomarkers, viral serologies or bacterial cultures and advanced imaging. Elevated troponins reflect myocardial injury, while inflammatory markers such as C-reactive protein (CRP) support the presence of active inflammation.^4^ Cardiac magnetic resonance imaging (CMR) is recognised as the gold standard due to its ability to detect myocardial oedema, fibrosis and inflammation.^5^

Treatment varies with cause and severity. Viral myocarditis is usually self-limiting, while bacterial cases often require antibiotics. Anti-inflammatory agents like colchicine and non-steroidal anti-inflammatories (NSAIDs) may reduce inflammation and recurrence.^6^^,^^7^ Follow-up imaging is essential to assess resolution and guide therapy.

This report details a rare instance of *Salmonella*-associated myocarditis in a young, immunocompetent, female patient. It illustrates the importance of considering atypical pathogens and individualised management on case-by-case basis.

## Case report

A woman in her early 20s presented to the emergency department with sharp retrosternal chest pain, which was exacerbated by movement and deep breathing and partially alleviated by leaning forward. This followed a week of watery diarrhoea, vomiting, abdominal cramps and systemic malaise, which had resolved 3 days prior. She denied fever, dyspnoea, palpitations, coryzal symptoms or recent travel. The patient had no significant past medical history or known cardiovascular conditions. She was not on any regular medications. No recent vaccinations or recent short courses of medications over the counter were reported.

Vital signs were within normal range, with a heart rate of 88 beats per minute, blood pressure of 118/72 mmHg, respiratory rate of 16 breaths per minute, and oxygen saturation of 98% on room air. Cardiovascular, respiratory and abdominal examinations were unremarkable.

Initial labs revealed elevated cardiac troponin I levels of 459 ng/L, with a peak value of 972 ng/L (Table 1). C-reactive protein (CRP) was elevated at 116 mg/L. Full blood count, renal and liver panels were normal. Stool culture detected *Salmonella enterica* subspecies *I* on polymerase chain reaction (PCR). Serological tests ruled out common viral causes of myocarditis, including coxsackievirus, adenovirus, Epstein–Barr virus, SARS-CoV-2, influenza type A and type B, cytomegalovirus, human immunodeficiency virus, hepatitis B and C, and parvovirus B19. *Mycoplasma pneumoniae, Coxiella burnetti* and *Borrelia burgdorferi* serologies were undetectable. Anti-streptolysin O (ASO) titres were negative, excluding streptococcal aetiology. Blood cultures were negative.

**Table 1:** Patient’s biochemistry results throughout hospital admission.

Laboratory value	Day 0	Day 2	Day 4	Day 6 (day of discharge)	Reference range
White blood cell (× 10^9^/L)	8.5	4.2	4.3	5.2	4.0–11.0
Neutrophils (× 10^9^/L)	6.7	1.3	1.6	2.3	2–7.5
Lymphocytes (× 10^9^/L)	0.8	2.2	2.0	2.1	1–4
Haemoglobin (g/L)	148	115	118	116	130–180
Platelets (× 10^9^/L)	291	307	315	348	150–400
Sodium (mmol/L)	138	140	140	141	133–146
Potassium (mmol/L)	3.3	4.0	4.0	4.5	3.5–5.3
Urea (mmol/L)	4.4	2.6	2.3	3.4	2.5–7.8
Creatinine (µmol/L)	93	55	56	61	49–90
Estimated glomerular filtration rate (eGFR – mL/min)	75	>90	>90	>90	90–120
Bilirubin (µmol/L)	3	3	5	3	0–20
Alanine transaminase (U/L)	28	17	14	14	0–55
Aspartate transaminase (U/L)	27	29	30	28	0–34
Troponin I (ng/L)	459 (972–6 hours later)	308	26	12	0–15
C-reactive protein (mg/L)	116	44	18	5.0	0–5

Table 1 displaying patient’s biochemistry from admission until discharge, showing improvement in inflammatory markers (C-reactive protein and White blood cell count) and serial troponin levels.

**Fig. 1:**
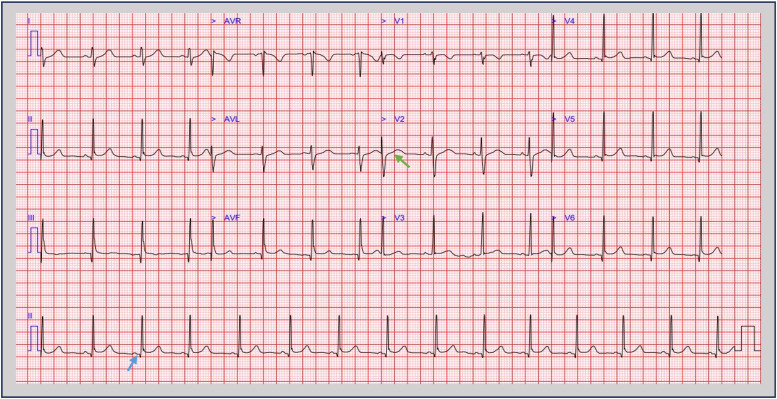
Electrocardiogram (ECG) taken during an episode of chest pain showing subtle ST segment elevation likely representing benign early repolarisation changes (green arrow) and PR segment depression (blue arrow) suggestive of myopericarditis.

**Fig. 2:**
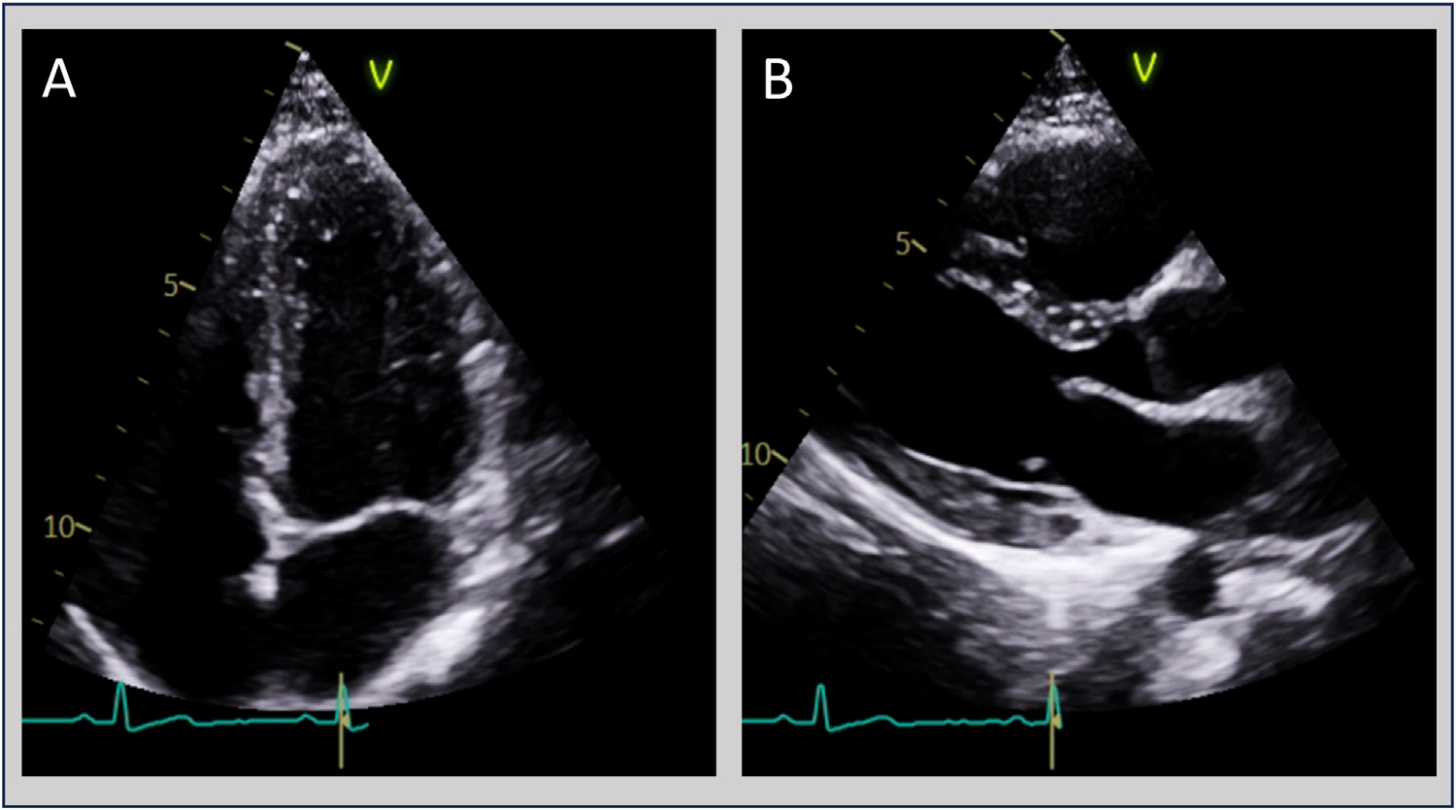
Echocardiogram images showing normal left ventricular cavity dimensions and wall thickness with no pericardial effusion observed. Image (A) showing the apical four-chamber view. Image (B) showing the parasternal long axis view.

Electrocardiography (ECG) revealed subtle concave ST-segment elevation and PR depression, consistent with myopericarditis (Fig. 1). Transthoracic echocardiography (TTE) demonstrated normal biventricular size and systolic function, with a left ventricular ejection fraction of 63%. There were no regional wall motion abnormalities, pericardial effusion or valvular pathology (Fig. 2).

**Fig. 3:**
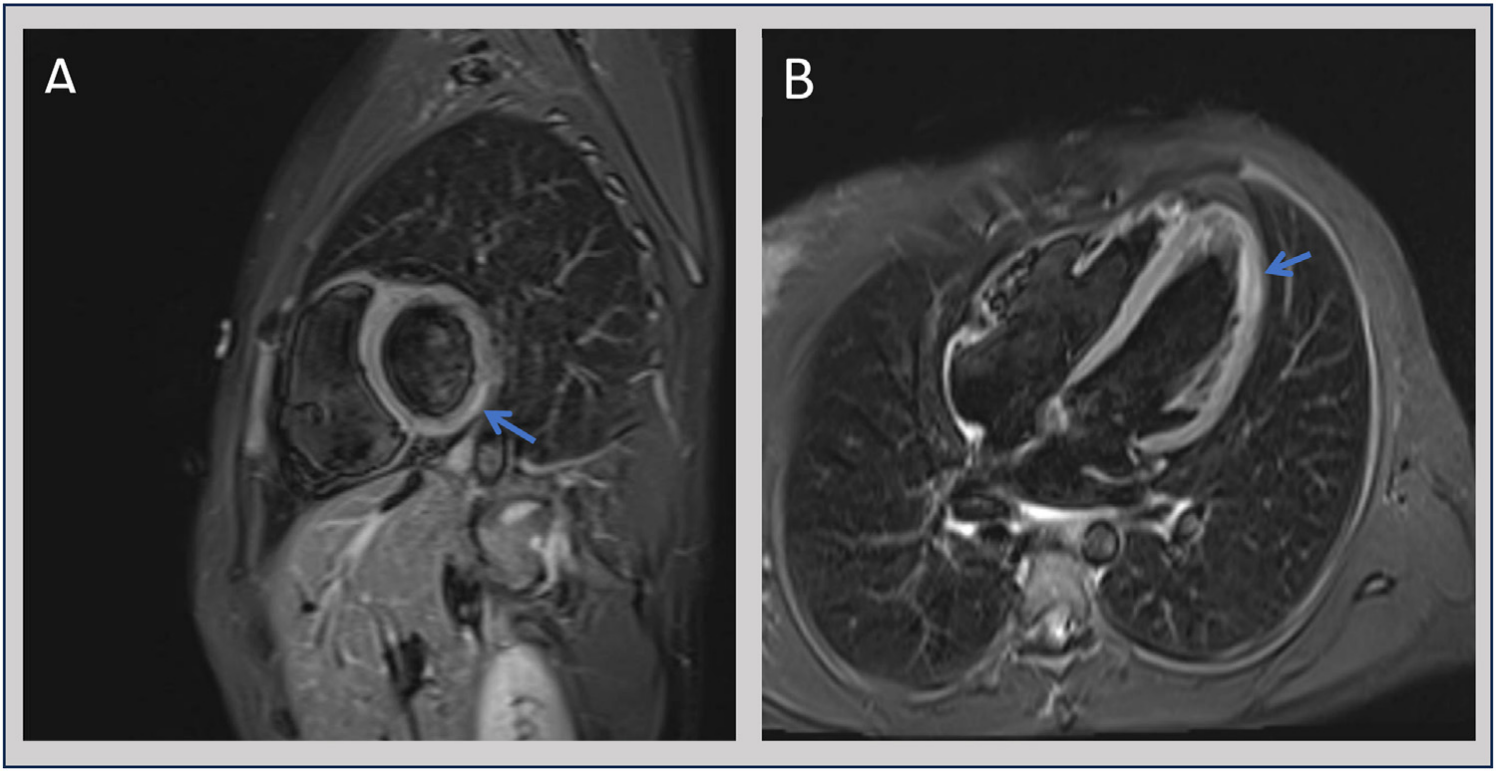
Cardiac magnetic resonance imaging (CMR) showing mild subepicardial oedema in the basal inferolateral myocardial wall (blue arrows) and corresponding late gadolinium enhancement (LGE) at the same segment, with no evidence of ventricular dilatation or fibrosis. Image A: short axis view. Image B: four-chamber view.

Cardiac magnetic resonance imaging (CMR) confirmed acute myocarditis with mild subepicardial late gadolinium enhancement (LGE) and oedema in the basal inferolateral wall. Biventricular systolic function was preserved, with ejection fractions of 63% and 57%, respectively. There was no evidence of chamber dilation or significant fibrosis (Fig. 3).

A diagnosis of acute myocarditis secondary to *Salmonella* gastroenteritis was made. Management was conservative, with colchicine 500 μg daily for 3 months. Antibiotics were not given due to clinical recovery and the absence of bacteraemia. Serial troponin I levels trended down and her chest pain resolved. She was discharged on day 6 with advice to avoid strenuous activity for 6 months, with a follow-up CMR planned in 3 months.

## Discussion

*Salmonella*-associated myocarditis is extremely rare, especially in immunocompetent individuals. This case highlights the diagnostic and therapeutic complexities of bacterial myocarditis in an immunocompetent individual and underscores the role of advanced imaging modalities in confirming the diagnosis.

The mechanism of *Salmonella*-associated myocarditis remains unclear, but is hypothesised to involve immune-mediated injury driven by systemic inflammatory responses to endotoxins rather than direct bacterial invasion of myocardial tissue.^3^ In the absence of bacteraemia, as seen in this case, the immune response likely predominates, driving a strong inflammatory reaction characterised by the release of pro-inflammatory cytokines.^8^ This, in turn, recruits inflammatory cells to the myocardium, leading to oedema and fibrosis. These observations align with prior case reports demonstrating that systemic infection is not a prerequisite for myocardial involvement.^9^

This patient’s pleuritic chest pain and elevated troponin following a diarrhoeal illness align with the expected clinical course of NTS-associated myocarditis. Troponins, while sensitive, are non-specific for myocarditis and require imaging correlation.^5^

ECG findings of ST elevation and PR depression support myopericardial involvement. Transthoracic echocardiography (TTE) is useful for assessing cardiac structure and ventricular function, which may be impaired in severe myocarditis; however, they may be normal in mild disease, as in this case.

CMR provides high-resolution myocardial tissue characterisation. In this case, subepicardial LGE and oedema were diagnostic.^10^

CMR is also useful for prognosis, with residual fibrosis or ongoing inflammation being associated with adverse outcomes, including arrhythmias and chronic cardiomyopathy.^11^ Endomyocardial biopsy (EMB) remains the histological gold standard, but is reserved for select cases due to its invasiveness and low sensitivity.^12^ In *Salmonella*-associated myocarditis, the diagnostic utility of EMB is further limited by its procedural risks and often yields non-specific findings. Histopathological findings in confirmed cases typically reveal histiocytic and lymphocytic myocardial infiltration; however, such diagnoses are often made post-mortem.^13^^,^^14^ Advances in non-invasive multimodal imaging, including echocardiography and CMR, now enable the detection of even subclinical myocarditis and have largely obviated the need for routine histological confirmation.

There are no standard guidelines for treating *Salmonella*-associated myocarditis, given its rarity. Colchicine was highly effective in controlling myocardial inflammation, as evidenced by the resolution of chest pain and declining troponin levels. The effectiveness of colchicine in myopericarditis is well supported^15^ in the ICAP and CORP trials, demonstrating efficacy in reducing symptoms and recurrence rates.^7^^,^^16^ Antibiotics were not given, as gastrointestinal symptoms had resolved with no evidence of systemic bacteraemia or sepsis, aligning with the recommendations for managing uncomplicated NTS infections.^17^

Early diagnosis and conservative treatment were associated with complete recovery. Follow-up CMR is important to confirm resolution and guide activity restrictions or further therapy.^11^

Although *Salmonella* is a bacterial pathogen, the role of antimicrobial therapy in *Salmonella*-associated myocarditis remains unclear, especially in immunocompetent patients without systemic infection. In our case, conservative management with colchicine and NSAIDs resulted in full clinical and biochemical recovery without complications or residual cardiac dysfunction, despite no antibiotics being administered. This contrasts with findings from Villablanca *et al,*^9^ where 90% of non-typhoidal *Salmonella* myocarditis cases were treated with antibiotics, although only 29% had confirmed bacteraemia. While that review generally supports antimicrobial use, it found no clear association between antimicrobial therapy and improved outcomes such as reduced mortality or hospital stay. Our patient exhibited no evidence of bacteraemia or sepsis, and the presentation was consistent with immune-mediated myocardial inflammation. In this context, withholding antibiotics was a cautious yet appropriate decision. This case supports an individualised approach to the pharmacological management of NTS myocarditis, with treatment guided by clinical judgement, imaging findings and the presence or absence of systemic infection, rather than routine antibiotic use.

There is a clear need for more robust data to inform the management of bacterial myocarditis. Prospective studies and case registries could help elucidate the pathophysiology of *Salmonella*-associated myocarditis and evaluate the efficacy of various therapies, including the roles of antibiotics and anti-inflammatories. Biomarkers predictive of disease severity and patient outcomes should also be investigated to guide risk stratification and individualised management strategies.

## Conclusion

This case illustrates the rare occurrence of myocarditis following *Salmonella* gastroenteritis in an immunocompetent host. It underscores the importance of considering bacterial causes in myocarditis, the value of cardiac MRI for diagnosis, and the potential for successful conservative management in select patients. Tailored, multidisciplinary care and close follow-up are essential for favourable long-term outcomes.

## CRediT authorship contribution statement

**Orestis Paschalis:** Writing – original draft, Data curation, Conceptualization. **Paul Njoku:** Writing – review & editing, Writing – original draft, Data curation, Conceptualization. **Amit K.J. Mandal:** Writing – review & editing, Supervision. **Constantinos G. Missouris:** Writing – review & editing, Supervision.

## Declaration of competing interest

The authors declare that they have no known competing financial interests or personal relationships that could have appeared to influence the work reported in this paper.
